# The analgesic effect of intraarticular OnabotulinumtoxinA in a female murine model of collagenase induced chronic degenerative monoarthritis

**DOI:** 10.1016/j.toxicon.2018.11.307

**Published:** 2018-11-22

**Authors:** Nicole Blanshan, Maren L. Mahowald, Christopher Dorman, Sandra Frizelle, Hollis E. Krug

**Affiliations:** a Rheumatology Department, Veterans Affairs Medical Center, Minneapolis, MN, United States; b Department of Medicine, University of Minnesota, Minneapolis, MN, United States

**Keywords:** Botox™, Osteoarthritis, Mouse, Pain, Neurotoxins

## Abstract

**Purpose:**

We previously reported the efficacy of intraarticular (IA) rimabotulinumtoxinB (BoNT/B) in a murine model of chronic degenerative arthritis pain. This study aimed to measure the analgesic effects of onabotulinumtoxinA (BoNT/A) on collagenase induced chronic degenerative arthritis joint pain.

**Methods:**

Chronic degenerative arthritis was produced by IA injection of 10 μl collagenase (Col) (10 IU) into the left knee of C57BL/6J female mice 4 weeks prior to pain assessment. IA BoNT/A was injected 3 days before testing. Arthritis pain was measured as evoked pain scores (EPS) and spontaneous pain behaviors with an advanced dynamic weight bearing (ADWB) device. EPS was a tally of fights and vocalizations exhibited in one minute with knee palpation. Percent body weight and percent time spent on each limb was quantified. All mice were 12 weeks old at the time of examination.

**Results:**

IA Col increased EPS and reduced ADWB measures of percent weight bearing on the left hind limb compared to naïve mice. BoNT/A treatment reduced EPS and increased weight bearing on the left hind limb. The improvements were not significant compared to the Col group. There was no significant difference in time spent on the left hind limb between any treatment groups. Forelimb ADWB measures of percent weight and time in arthritic mice significantly increased compared to nonarthritic animals. Treatment with BoNT/A in the arthritic limb decreased this offloading; however, statistical analysis only showed significance in weightbearing.

**Conclusion:**

IA Col monoarthritis increased evoked and spontaneous pain behaviors in female mice after four weeks. Treatment with IA BoNT/A decreased pain behaviors but only forelimb weight bearing showed a significant improvement. This led us to conclude that treatment with BoNT/A is not an effective analgesic for the treatment of chronic degenerative knee arthritis in murine models.

## 1. Introduction

Osteoarthritis (OA) is the most commonly diagnosed chronic disease, affecting approximately 27 million adults in the United States (Hootman et al., 2016; Lawrence et al., 2008). By the year 2030 this number is expected to surpass 67 million (Hootman and Helmick, 2006). The most debilitating OA symptom affecting patients is pain (Malfait and Schnitzer, 2013; Neogi, 2013). Living with chronic pain poses negative personal and societal ramifications. Patients may experience difficulties completing daily activities, routine needs and personal care (Centers for Disease Control and Prevention, 2010). Among working age adults, 30.6% reported serious work limitations (Theis et al., 2007). In a 2010 Medical Expenditures Panel Survey, the estimated cost from absenteeism due to OA pain was $11.6 billion annually (Kotlarz et al., 2010). Further cost analyses for the United States showed $162 billion in combined direct (medical expenditures) and indirect costs (lost wages). This accounts for 2% of the annual gross domestic product (Kotlarz et al., 2010; Yelin et al., 2007).

There is no cure for osteoarthritis, thus treatment goals are focused on pain management. A combination of pharmacological and non-pharmacological practices is commonly utilized. Pharmacological options include oral (systemic), intraarticular, and surgical therapies. Non-pharmacological options include: education, exercise, weight reduction, acupuncture, and joint protection (Wenham and Conaghan, 2013). An examination of these practices found insufficient joint pain relief, intolerable drug side effects and interactions, and minimal compliance with integrative therapies (Conaghan, 2012; Doherty et al., 2011; Zhang et al., 2010). In the most degenerative cases surgical treatment may be used. Several analyses concluded alleviation of pain from surgical therapies were no greater than those obtained from placebo (Kirkley et al., 2008; Mosely et al., 2002; Zhang et al., 2010). Total joint replacement has significant risk and is inappropriate for individuals with comorbidities but can alleviate pain. Therefore, there is an important unmet need for safe, effective pain treatment.

Generation and maintenance of chronic arthritis pain is complex. Nociceptive articular pain originates at the site of disease or injury. The nociceptive input is a response to noxious mechanical, thermal, or chemical stimuli. Local inflammatory mediators (H^+^, Glutamates, Bradykinin, cytokines, and chemokines) and neuropeptides released in the periphery by efferent neurogenic signals amplify and maintain arthritis pain by lowering the threshold for nociceptor activation (Miller et al., 2014; Schaible et al., 2006). Local release of neuropeptides such as substance P and calcitonin gene-related peptide from articular sensory nerve endings causes vasodilatation, extravasation of plasma, chemotaxis of macrophages, and mast cell degranulation (Schaible et al., 2006). Given this peripheral sensitization, we hypothesize arthritis pain may be treated effectively by intraarticular neurotoxins.

Botulinum neurotoxins (BoNT) are comprised of seven different serotypes that mechanistically inhibit the release of neuronal signal chemicals from sensory nerves which contribute to peripheral sensitization. All serotypes act by hindering SNARE-associated exocytosis of acetylcholine and other neuropeptides into the synaptic cleft (Dolly and Aoki, 2006; Oh and Chung, 2015). This mechanism begins with the endocytosis of BoNT, comprised of a heavy and light chain, into the presynaptic terminal via presynaptic membrane vesicle (Zanetti et al., 2015). The light chain is then translocated into the cytosol and acts as a protease, cleaving different SNARE proteins (Fischer and Montal, 2007). Without SNARE proteins, facilitated transportation of neuronal signal chemicals across the presynaptic membrane into the synaptic cleft does not occur (Pirazzini et al., 2017). Several recent studies have shown that BoNT may not stay localized in the axon terminal but also undergoes axonal transport (Favre-Guilmard et al., 2017; Marinelli et al., 2012; Restani et al., 2011). The main differences between the seven BoNT serotypes are based on affinities and which SNARE protein (s) they cleave (Aoki and Guyer, 2001; Mense, 2004; Pellizzari et al., 1999).

In our earlier studies, we examined the analgesic effects of rimabotulinumtoxinB (BoNT/B) in an OA murine model. Anderson et al. used joint palpation to measure evoked pain behaviors and visual gait analysis to measure spontaneous pain behaviors. Both significantly improved with IA BoNT/B treatment (Anderson et al., 2010). Our group concluded that further analysis of Botulinum neurotoxins was necessary.

OnabotulinumtoxinA (BoNT/A) differs from BoNT/B in several ways. The serotypes cleave different SNARE proteins; BoNT/A cleaves SNAP-25 and BoNT/B cleaves VAMP/synaptobrevin (Pellizzari et al., 1999; Verderio et al., 2007). There have been several preclinical studies showing that BoNT/A inhibits the release of local nociceptive neuropeptides (Aoki, 2005, 2008; Cui et al., 2004). And BoNT/A is the only serotype with FDA approval for peripheral applications to treat pain (Burstein et al., 2014).

We hypothesized that IA BoNT/A could reduce pain behavior measures and produce analgesia in a collagenase induced OA murine model. The purpose of this study was to measure the analgesic response produced by BoNT/A in female mice with chronic degenerative monoarthritis pain using evoked and spontaneous pain behaviors. This animal model of OA was chosen because it is a well-established model and has been used previously to study IA BoNT/B.

## 2. Material and methods

### 2.1. Animals

Studies were performed on 12-week-old female C57BL/6J mice (Jackson Laboratories, Bar Harbor, ME). Healthy specific pathogen free (SPF) mice were delivered at 6–7 weeks and acclimated to their housing for one week prior to experiments. Mice were housed in SPF conditions in standard polycarbonate cages with water and standard rodent diet ad libitum. Environmental conditions were held constant at: 21 ± 3 °C, 33 ± 1% humidity, and 12-h light/dark cycle. All animal procedures and protocols were approved by the Minneapolis Veterans Affairs Health Care System (VAHCS) Institutional Animal Care and Use Committee and conformed to the “Guide for the Care and use of Laboratory Animals” (The National Academic Press, USA). Mice were maintained in the Animal Research Facility at the Minneapolis VAHCS, an Association for Assessment and Accreditation of Laboratory Animal Care (AAALAC) approved facility. Female mice were used with the future goal of sex studies in mind. Estrous cycles were not evaluated based on the work of Meziane et al. showing pain behaviors are not affected by estrous phases in female C57BL/6J mice.

### 2.2. Study design

**Fig. 1.**
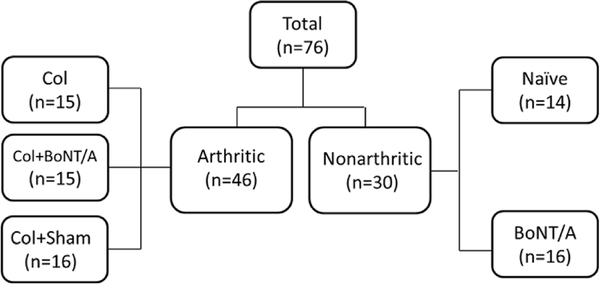
Overview of Study. A total of 76 animals were studied in six groups of 14–16 mice. Forty-six arthritic animals were separated into three groups: arthritic (Col), treated arthritic (Col+BoNT/A) and arthritic plus puncture control (Col+Sham). Thirty nonarthritic animals were separated into two groups: uninjected nonarthritic control (naïve) and treatment control (BoNT/A).

A total of seventy-six animals were studied. Mice were randomly allocated to the various experimental groups. Collagenase was used to induce monoarthritis in 46 mice. Of these, fifteen arthritic mice received IA BoNT/A treatment, 16 arthritic mice were given a sham injection (puncture control) and 15 were untreated (arthritis control). Thirty nonarthritic mice served as controls: 14 mice did not receive IA injection (naïve) and 16 received IA BoNT/A (treatment control) (Fig. 1). Group numbers were determined by power calculation to detect a 25% difference with a power of 80% and a significance level of 0.05.

### 2.3. Injections

Animals that received IA injections were chosen in random order and anesthetized with isoflurane in oxygen (induction and maintenance 3%, 1 L/minute) for 3 ± 1 min. This anesthesia has been determined to be safe, provides rapid induction and recovery with minimal adverse effects. IA injections were performed on the left knee. The injection site was shaved and prepared with 0.75% povidone-iodine scrub prior to injection. A 30-gauge needle was fitted with a customized sheath that limited the depth of needle penetration to 2.5 mm. The injection was performed through the midline of the patellar tendon just inferior to the patella to ensure accurate entry into the articular space. Chronic arthritis was produced in the left knee of 8-week-old female mice by IA injection of 10 IU of Type IV collagenase (Worthington Biomedical Corporation, Lakeville, NJ Cat#LS004210) in 10 μl of normal saline (van der Kraan et al., 1990). Pain behavior testing was performed four weeks’ post injection. The uninjected right knee served as the internal nonarthritic control. IA onabotulinumtoxinA (BOTOX™, Allergan, Plc, Dublin, Ireland) (0.02 U/knee in 5 μl of sterile saline) was injected three days prior to pain behavior testing in arthritic and nonarthritic animals. The dose and timepoint were determined based on previous ranging studies (data not shown). The dose was significantly below the LD50 for mice for this compound. Sham injections were performed as described above on the left hind arthritic limb. Animals were observed during recovery until they were ambulatory at which time they were returned to the home cage.

### 2.4. Measurement of evoked joint pain

Evoked joint pain is the experimental correlate of palpable joint tenderness. This was quantified by counting the number of fights and vocalizations elicited by the animal during one minute of repeated palpation of the knee. Fights were defined as noticeable flinches or escape attempts. Vocalizations were defined as audible sounds emitted by the animal. Prior to these studies a single blinded examiner was trained using a “Palpometer” (Palpometer Systems, Inc. Victoria, B.C.) to reproducibly exert the same force (1100 gf/cm2 = 15.6 PSI) to the medial and lateral sides of the knee (palpation). In this experiment the Palpometer was not used on the mice. Testing was performed during the morning hours for each group. The blinded examiner removed an animal at random order from the home cage and held it in one hand while palpating the knee with their opposite hand. Intermittent pressure with thumb and index finger was applied to each side of the joint every second for one minute. The pain responses during palpation were counted and totaled by a second observer for the evoked pain score (EPS). The normal right knee was always examined first. In preliminary experiments with tenderness testing, our group found slightly elevated tenderness scores in whichever knee was examined second. This phenomenon, known as wind up, has been well documented. Most recently, Ziv et al. found naïve mice submitted to low intensity stimulation exhibited heightened sensitivity in subsequent response tests.

### 2.5. Measurement of spontaneous pain behavior

Advanced Dynamic Weight Bearing (ADWB) (Bioseb In Vivo Research Instruments, Vitrolles, France) was used to quantify spontaneous nociceptive behavior in mice (Griffioen et al., 2015). The device was a 4.5 × 4.5 × 8.25 in^3^ plexiglass chamber with a floor consisting of 1936 pressure sensors. The sensors were calibrated prior to each experiment by placing a 2680-g weight on the pad and adjusting the individual sensors to a normalized mean. The animal’s weight was recorded in the software before placing the mouse in the chamber for a five-minute period. To ensure spontaneous behavior, mice were not acclimated to the chamber before the collection of data and were allowed to freely move once inside the chamber. All activity was recorded with synchronized video and sensor data, which was relayed to the ADWB software. The software partitioned the five-minute video into analyzable and non-analyzable segments. Segments were considered analyzable when at least one stable zone was detected. A zone was created when sensors detected ≥2g on one sensor with a minimum of one adjacent sensor detecting ≥1g and when the weight distribution of a zone was stable for more than 0.38 s. Zones that met these minimal criteria were presented to the observer for assignment as right hind, left hind, right fore, left fore, or both forepaws per the video and scaled map of activated sensors. Validation of stable zones occurred as the observer worked through the analyzable segments and defined a paw(s) to each stable zone. A minimum of 1.5 min of analyzable time was required to meet analysis thresholds. Analyzed segments were added and averaged for output per paw(s). The ADWB software then calculated the percentage of analyzed time spent and percentage of body weight (g) placed on each of the five limb options. At the end of the study period animals were euthanized by CO2 inhalation followed by thoracotomy. Tissues were preserved for histology.

### 2.6. Histologic examination of normal and arthritic knees

The left hind limbs of one mouse in the following conditions: naïve, arthritic, and treated arthritic mice, were examined for histologic evidence of degenerative arthritis following conclusion of the study. Extremities were dissected. Articular specimens were fixed in 10% buffered formalin for 24 h and decalcified in Decal Stat™ Decalcifier (StatLab Medical Pro, McKinney, Texas) for 24 h before paraffin embedding. Paraffin-embedded blocks were serially sectioned into one hundred 4 μm sections. Twenty representative slides per mouse were stained for cartilage proteoglycans with Safranin O/Fast Green staining. OA severity was quantified using the Osteoarthritis Research Society International (OARSI) scoring system (Glasson et al., 2010). Stained sections were analyzed by a blinded researcher using the 0–6 subjective system outlined in Table 1. Individual scores for each treatment were averaged for the reported grade.

### 2.7. Statistical methods

EPS and ADWB results for the five independent groups (naïve, BoNT/A, Col, Col+Sham, and Col+BoNT/A) were compared using an ANOVA one-way analysis of variance. Dunnett’s post hoc *t*-test was used for comparing groups whenever the overall analysis of variance was significant. In all cases, differences were considered significant when *P* values were less than 0.05. Throughout the manuscript the term significant is used to describe statistical significance.

## 3. Results

### 3.1. Measurement of evoked pain behaviors

**Fig. 2.**
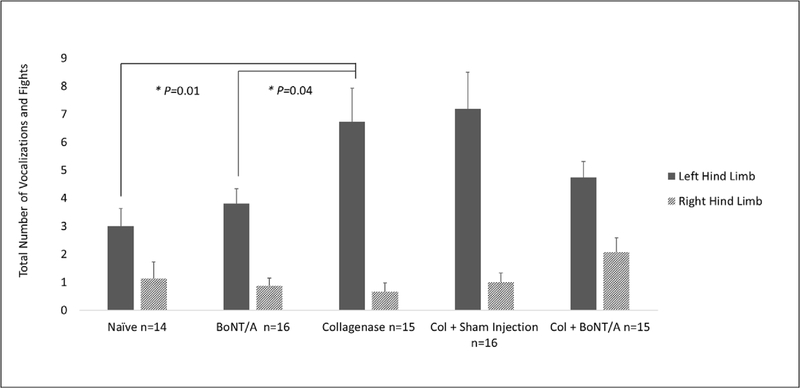
Evoked pain scores for left (solid column) and right (striped column) hind limbs. Right hind limbs served as internal controls and did not undergo any IA injections. Measurements were taken in the following conditions: uninjected naïve, treatment only (BoNT/A), arthritic (Col), arthritic puncture control (Col+Sham), and treated arthritic (Col+BoNT/A). One-way analysis of variance comparing evoked pain in all treatment groups found a significant difference (P = 0.0065). To explore differences between groups, post hoc analysis using Dunnett’s *t*-test was performed. Analysis found that the mean EPS was significantly higher (6.73, SEM 1.19) in mice with monoarthritis than naïve (3.0, SEM 0.62) (P = 0.011). Treatment with BoNT/A decreased EPS to 4.73 (SEM 0.58) in the arthritic limb. This failed to be significantly different from arthritic. The treatment only group (BoNT/A) exhibited significantly lower EPS (3.81, SEM 0.52) than arthritic mice (P = 0.043). The arthritic puncture control group (Col+Sham) had slightly elevated EPS (7.20, SEM 1.30) compared to arthritic but did not show significance. EPS in the contralateral knee fell slightly from a baseline mean of 1.14 (SEM 0.58) in all treatment groups except Col+BoNT/A (2.07, SEM 0.52) which increased slightly. These values failed to show any significant difference when compared to the right hind limb of arthritic mice. (* = P < 0.05).

EPS from the left hind knee of naïve mice was 3.0 fights and vocalizations (SEM 0.62). This score significantly increased to 6.73 (SEM 1.19, *P* = 0.011) in mice with Col induced arthritis. BoNT/A treatment of arthritic left hind knees reduced EPS to 4.73 (SEM 0.58); however, this was not a significant difference when compared to arthritic mice (Fig. 2).

### 3.2. Measurement of spontaneous pain behaviors

#### 3.2.1. Weight

**Fig. 3.**
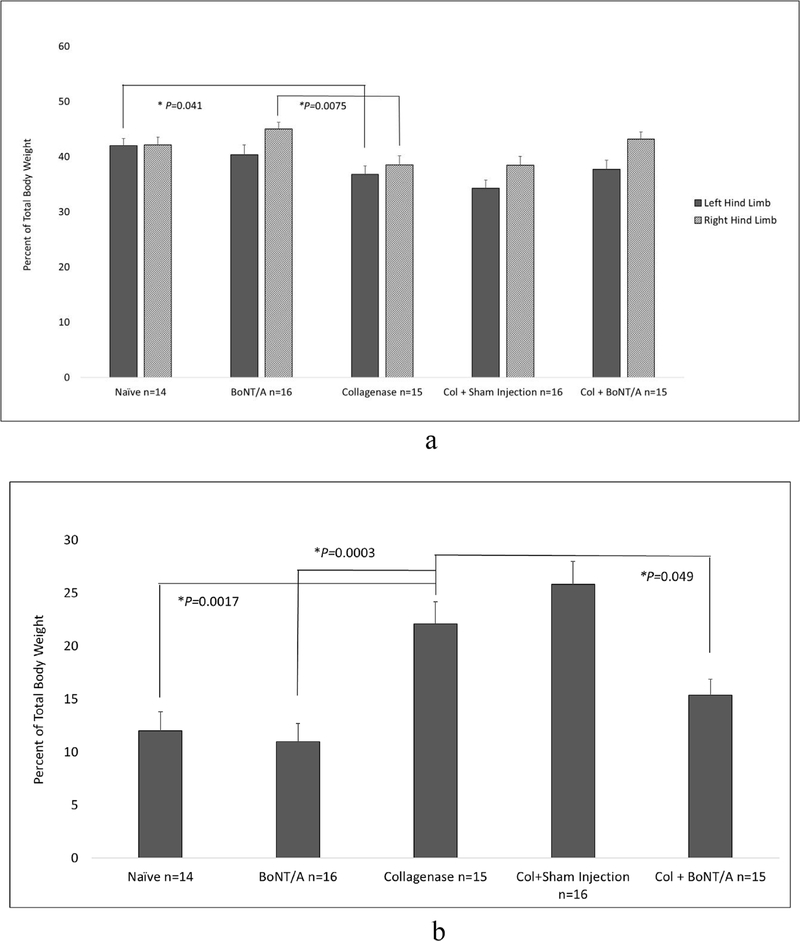
**a.** Spontaneous pain behavior measured by total percent body weight on the left (solid column) and right (striped column) hind limbs. One-way analysis of variance comparing weight bearing in the left hind limbs of all treatment groups found a significant difference (P = 0.0084). Post hoc analysis found that mice with monoarthritis bore significantly less weight (36.79%, SEM 1.52) on the affected limb than naïve (41.98%, SEM 1.33) (P = 0.041). Treatment with BoNT/A increased weight bearing to 37.73% (SEM 1.61) on the left hind limb but was not significantly different when compared with arthritic or naïve mice. The treatment control group (BoNT/A) (40.4% SEM 1.8) and the puncture control group (Col+Sham) (34.3%, SEM 1.5) showed no significant difference in weight bearing on the left hind limb compared to arthritic mice. Statistical difference in weight bearing on the contralateral, nonarthritic right hind limb was not found when comparing groups to arthritic mice. Except in the treatment only group (BoNT/A) (45.0%, SEM 1.23) which bore significantly more weight on the right hind limb than arthritic mice (P = 0.0075). (* = P < 0.05). **3b**. Spontaneous pain behavior measured by total percent body weight placed on the forelimbs. One-way analysis of variance comparing spontaneous pain of all treatment groups found a significant difference (P < 0.0001). Post hoc analysis found that mice with monoarthritis in the left hind limb bore significantly more weight (22.07%, SEM 2.10) on their forelimbs than naïve (12.01%, SEM 1.77) (P = 0.0017). Treatment with BoNT/A significantly decreased this weight distribution to 15.36% (SEM 1.53) (P = 0.049). The treatment only control group (BoNT/A) bore significantly less weight on the forelimbs (10.99%, SEM 1.70) than arthritic mice (P = 0.0003) which would be expected for a treatment without significant toxicity. Forelimb weightbearing in the arthritic puncture control group (Col+Sham) (25.8%, SEM 2.18) showed no significant difference compared to arthritic mice. (* = P < 0.05).

Weight bearing on the left hind limb of naïve mice was 42.0% (SEM 1.3) of the total body weight. Mice with Col induced arthritis significantly decreased weight bearing to 36.79% (SEM 1.52) of the total body weight compared to naïve mice (*P* = 0.041). BoNT/A treated arthritic mice increased weight bearing to 37.73% (SEM 1.61); however, there was no significant difference when compared to arthritic mice (Fig. 3a).

Forelimb weight bearing in naïve mice was 12.01% (SEM 1.77) of the total body weight. Mice with Col induced arthritis in the left hind limb significantly increased forelimb weight bearing to 22.07% (SEM 2.10) (*P* = 0.0017). BoNT/A treated arthritic mice significantly decreased forelimb weight bearing to 15.36% (SEM 1.53) (*P* = 0.049) total body weight compared to mice with Col induced arthritis (Fig. 3b).

#### 3.2.2. Time

**Fig. 4.**
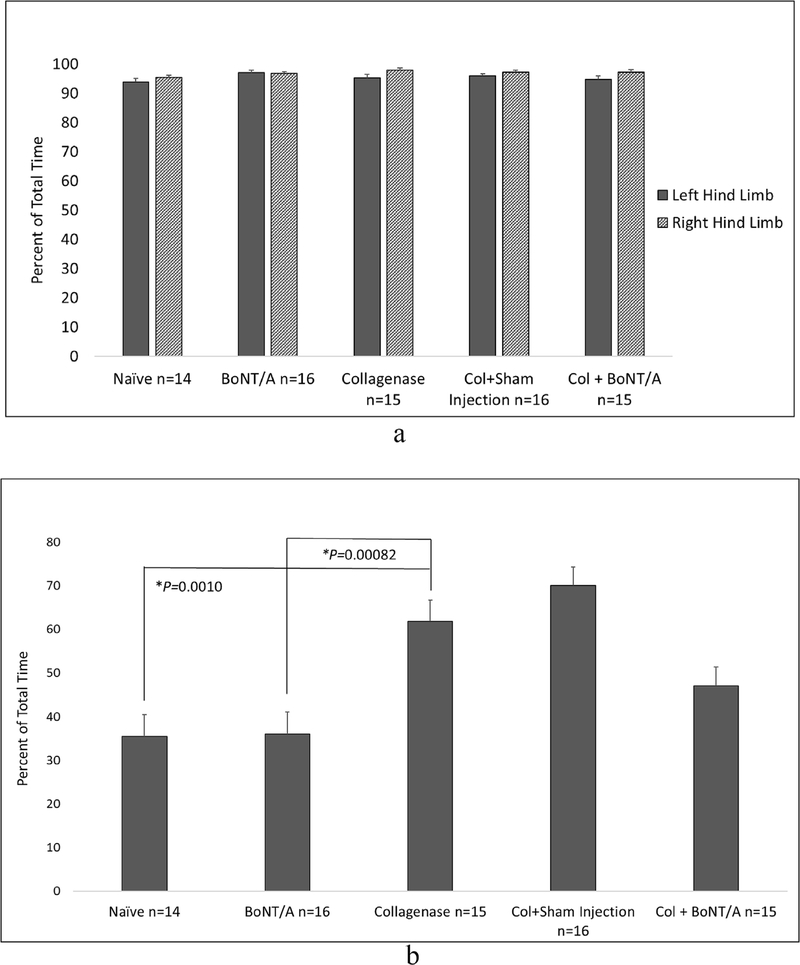
a. Spontaneous pain behavior measured by total percent time spent on the left (solid column) and right (striped column) hind limbs. One-way analysis of variance comparing percent time spent on the left hind limbs of all treatment groups found no significant difference. The same was done for the right hind limbs. No significant difference was found between any groups. (* = P < 0.05). **4b**. Spontaneous pain behavior measured by total amount of time spent on the forelimbs. One-way analysis of variance comparing spontaneous pain behaviors of all groups found a significant difference (P < 0.0001). Post hoc analysis found that mice with monoarthritis in the left hind limb spent significantly more time (61.79%, SEM 4.92) on their forelimbs than naïve (35.42%, SEM 5.0) (P = 0.001). Treatment with BoNT/A decreased this percent time to 47.07% (SEM 4.32) which was not significantly different when compared with arthritic mice but also was not different from naive. The treatment only control group (BoNT/A) spent significantly less time on the forelimbs (35.94%, SEM 5.14) than arthritic mice (P = 0.00082) which would be expected. The arthritic puncture control group (Col+Sham) spent slightly more time (70.07%, SEM 4.21) on the forelimbs than arthritic mice but it was not significant. (* = P < 0.05).

The percent of total time spent on the left hind limbs of naïve mice was 93.83% (SEM 1.20). This increased to 95.31% (SEM 1.17) in mice with Col induced arthritis. Treatment with BoNT/A slightly decreased the total time spent on the arthritic left hind limb to 94.67% (SEM 1.23). No significant difference was observed between any groups (Fig. 4a).

Interestingly, the percent of total time spent on the forelimbs was significantly increased to 61.79% (SEM 4.92) in arthritic mice compared to 35.42% (SEM 5.0) in naïve (*P* = 0.0017). BoNT/A treated arthritic mice decreased the percentage of time spent on the forelimbs to 47.07% (SEM 4.32). This difference was not significant when compared to arthritic mice (Fig. 4b).

#### 3.2.3. Normal right limb controls

Throughout all stages of this study, the contralateral right limb was a normal, noninjected internal control. EPS of the right knees showed no significant differences between any of the groups. The ADWB measures for mean weight and time of the nonarthritic right hind limb were analyzed. Only the BoNT/A group (44.99%, SEM 1.23) showed a significant increase in weight bearing compared to arthritic mice (38.49%, SEM 1.67) (*P* = 0.0075). (Figs. 3–4).

#### 3.2.4. Neurotoxin and puncture controls

Treatment control groups included 16 naïve mice receiving BoNT/A only and 16 arthritic mice receiving sham injections into the left knee. Animals were examined three days following either BoNT/A or sham injection and compared with untreated arthritic mice. There were no significant changes in pain behavior scores from the left hind limb of Col+Sham compared to arthritic mice. EPS were significantly less in BoNT/A only (3.81, SEM 0.52) compared to arthritic mice (6.73, SEM 1.19, *P* = 0.043). Compared to naïve (3.0), this group (BoNT/A only) had slightly elevated EPS, but no significance was observed. Weight bearing and time spent on the left hind limbs of BoNT/A only and arthritic mice were not significantly different (Figs. 3a–4a).

BoNT/A only mice bore significantly less weight (10.99%, SEM 1.70) and spent significantly less time (35.94%, SEM 5.14) on the forelimbs compared to arthritic mice (22.07%, SEM 2.10, *P* = 0.0003, 61.79%, SEM 4.92, *P* = 0.00082, respectively) (Figs. 3b–4b).

#### 3.2.5. Histological examination of knees

**Fig. 5.**
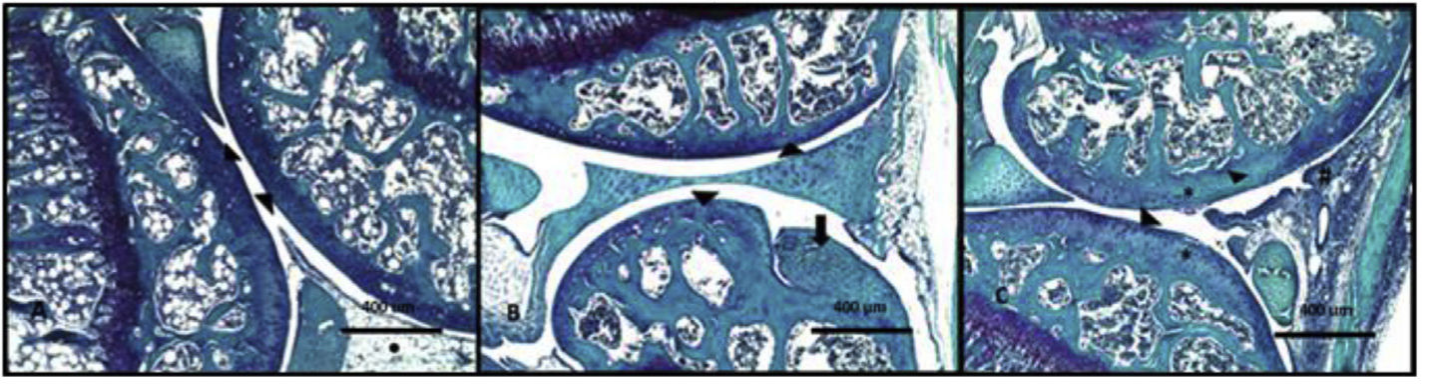
**(A–C)**. Safranin O/Fast Green stained 4 μm thick sections. Magnification 10x. (A) Uninjected naive left knee. Normal appearing articular cartilage (arrowhead) and synovium (circle). (B) Col injected left knee. Proteoglycan loss of articular cartilage (arrowhead) consistent with early degenerative arthritis. Cruciate ligament (arrow). (C) Col+BoNT/A injected left knee. Proteoglycan loss of articular cartilage (arrowhead), irregularity and fissuring of the superficial cartilage layers (*) and minor inflammation at injection site (#). BoNT/A injection did not improve or worsen the degenerative changes in the arthritic knee.

After sacrifice, the left knees of naïve, Col, and Col+BoNT/A mice were examined for histological evidence of degenerative arthritis. Safranin O/Fast green staining of naïve left knees showed only minor histologic alterations in the cartilage, meniscus and synovium with minor loss of proteoglycan (PG) staining of focal areas of articular cartilage. Staining of arthritic left knees showed more extensive PG loss in articular cartilage with irregularity and fissuring. There was minor inflammation of the arthritic knees that had been injected with BoNT/A 3 days prior to sacrifice. BoNT/A injection did not worsen the degenerative changes of cartilage in Col injected mice. Using the OARSI grading scale for murine osteoarthritis, arthritic knees and treated arthritic knees scored a maximum grade of 2. Naïve knees scored 0.5. (Fig. 5A–C).

## 4. Discussion

This study demonstrated that Col induced chronic degenerative knee monoarthritis produced measurable evoked and spontaneous nociceptive behaviors that were used to measure the possible analgesic effects of BoNT/A. EPS significantly increased from 3.0 to 6.73 in mice with Col induced arthritis in the left hind knee (Fig. 2). Weight bearing on the left hind limb in this group significantly decreased from 41.98% to 36.79% of the total body weight (Fig. 3a). When the arthritic limb was treated with BoNT/A, EPS decreased to 4.73 and weight bearing increased to 37.73%. However, this was not a significant improvement for either pain behavior measure (Figs. 2 and 3a). Percent time spent on the hind limbs was not a sensitive measure of arthritis pain for this model and therefore an analgesic affect could not be observed (Fig. 3b).

The limited changes in pain behavior in the hind limbs led us to more closely examine the forelimbs for evidence of analgesia using ADWB. Results showed a significant increase in percent weight bearing and time spent on the forelimbs of mice with Col induced monoarthritis. Treating the arthritic limb with BoNT/A decreased weight bearing on the forelimbs. Although time spent on the forelimbs decreased towards normal, we only observed a significant improvement in the weight bearing measure (Figs. 3b and 4b). While BoNT/A treated arthritic mice showed a decreased pain response in both evoked and spontaneous pain measures, it did not do so in a significant manner, apart from the forelimb weight distribution.

In earlier works studying BoNT/B, we utilized EPS to measure evoked pain and visual gait analysis to measure spontaneous pain behavior. EPS in the left hind knee of arthritic mice was 7.23 (SEM 0.953). Treatment with BoNT/B decreased this score to 3.58 (SEM 0.816). This was a 49.50% improvement. Treatment with BoNT/A also decreased evoked pain behaviors but a smaller improvement was observed 34.90%. The amount of improvement observed with BoNT treatment is similar but slightly less with BoNT/A. Explanation for this can be found in differences in binding mechanisms of the serotypes to the presynaptic membrane. It is known that BoNT/A binds to synaptic vesicle proteins SV2 and BoNT/B binds to synaptotagmin (Syt-I and II) (Berntsson et al., 2013; Rummel, 2017; Strotmeier et al., 2014). Recent work by Stern et al. shows that the binding of BoNT/B to its membrane protein receptors is one of higher affinity, thus less risk of dissociation before uptake into the neuron. The binding strength lies with a hydrophobic loop located on the heavy chain of the molecule. It mediates the formation of a highly stable BoNT-receptor complex comprised of the protein receptor, gangliosides and lipids (Stern et al., 2018).

For the purposes of this manuscript, it is important to note that these two studies were done years apart under similar but different conditions (location, time of year, observers) and should not be directly compared. A head-to-head comparison would be needed to definitively decide which is better and is beyond the scope of this paper.

Comparing spontaneous pain behaviors in arthritic animals treated with botulinum toxins elicited a 43% improvement with BoNT/B. ADWB parameters improved as follows with BoNT/A treatment: weight bearing on the arthritic hind limb 2.51%, weight bearing on the forelimbs 35.87%, time spent on the arthritic hind limb 0.67% and time spent on the forelimbs 29.04%. The methods used in the BoNT/B study were a semiquantitative subjective visual gait analysis and quasi-spontaneous gait analysis, which were utilized prior our use of ADWB. Our group determined these methods to be less reliable (Dorman et al., 2013). Analysis with ADWB is more reproducible, sensitive and it shows that female mice may not consistently offload to the predicted contralateral hind limb. Our data suggests mice also depend on the forelimbs as the joint stability decreases and pain increases under arthritic conditions.

For example, the analgesic control group (BoNT/A only) in the current work, slightly decreased weight bearing on the left hind limb compared to naive. Simultaneously, the right hind limb of this group seemed to have a compensatory increase in weight bearing. When compared to arthritic mice, the BoNT/A only group bore significantly more weight on the right hind limb. This shows partial offloading. Interestingly, arthritic mice decreased weight bearing in both the right and left hind limbs (Fig. 3a). In the BoNT/A group, this is most likely due to discomfort associated with the injection as we do see a slight insignificant increase in EPS as well. In the more painful arthritic state, mice reduced weight bearing on both hind limbs, while forelimb measures significantly increased (Fig. 3b). This is an important observation and reminds us that in quadrupeds there may be multiple adaptive gait behaviors in response to pain (Anderson et al., 2010; Malfait et al., 2010; Mogil, 2009).

This is different than what we have seen previously in inflammatory models of arthritis and in males (unpublished data), suggesting that severity of pain and sex of mice may influence these adaptive behaviors (Abdullah et al., 2016; Bert et al., 2016; Lam et al., 2004; van der Kraan et al., 1990). Therefore, it is important to use both evoked and spontaneous pain behavior measures to analyze analgesic responses to articular pain. Future studies will need to take these observations into account.

This work does not support our hypothesis that BoNT/A treatment reduces evoked and spontaneous pain behaviors in female mice with chronic degenerative arthritis. The only significant pain reduction observed was in forelimb weight bearing. It is possible that in this arthritis pain model, the pain is not as severe as in other models. Inflammatory arthritis pain is amplified by release of inflammatory mediators in the painful joint. BoNT is anti-inflammatory in that it inhibits release of proinflammatory neuropeptides. The degenerative model of arthritis pain used in this work likely produces less inflammation; hence, the BoNT/A has a less potent effect.

Our study has several potential limitations. The groups were small so slight differences may not have been detected statistically. The results cannot be extrapolated to other models of experimental arthritis. The use of only a single time point of arthritis duration and treatment may have missed the point of greatest pain and efficacy. Histologically, this was very mild degenerative arthritis. Evaluation of efficacy of BoNT/A in more advanced degenerative arthritis pain may be informative. We were not able to directly compare responses from BoNT/B and BoNT/A treated mice due to differing analysis methods of spontaneous pain behaviors.

Despite these technical hurdles, the most notable strengths of this study are the use of standardized measures that reproducibly quantitate spontaneous and evoked pain behaviors. These findings provide evidence to move forward with investigation of additional neurotoxins for arthritis pain. The advantage of using animal models for these studies is that we can examine directly the effect of these neurotoxins on the nervous system. This will be a major advantage as we move forward with studies of combination IA toxins for intractable arthritis pain.

## Figures and Tables

**Table 1:** Quantitative scoring system for grading stained histological slides.

Grade	Osteoarthritic damage
0	Normal
0.5	Loss of Safranin-O without structural changes
1	Small fibrillations without loss of cartilage
2	Vertical clefts down to the layer immediately below the superficial layer and some loss of surface lamina
3	Vertical clefts/erosion to the calcified cartilage extending to < 25% of the articular surface
4	Vertical clefts/erosion to the calcified cartilage extending to 25–50% of the articular surface
5	Vertical clefts/erosion to the calcified cartilage extending to 50–75% of the articular surface
6	Vertical clefts/erosion to the calcified cartilage extending to > 75% of the articular surface
